# DNA Damaged Induced Cell Death in Oocytes

**DOI:** 10.3390/molecules25235714

**Published:** 2020-12-03

**Authors:** Jakob Gebel, Marcel Tuppi, Nicole Sänger, Björn Schumacher, Volker Dötsch

**Affiliations:** 1 Institute of Biophysical Chemistry and Center for Biomolecular Magnetic Resonance, Goethe University, 60438 Frankfurt, Germany; gebel@bpc.uni-frankfurt.de (J.G.); marcel.tuppi@crick.ac.uk (M.T.); 2 Department for Gynecological Endocrinology and Reproductive Medicine, University Hospital of Bonn, Venusberg-Campus 1, 53217 Bonn, Germany; Nicole.Saenger@ukbonn.de; 3 Institute for Genome Stability in Aging and Disease, Cologne Cluster of Excellence in Cellular Stress Responses in Aging-Associated Diseases (CECAD) Research Center, and Center for Molecular Medicine, University of Cologne, Joseph-Stelzmann-Str. 26, 50931 Cologne, Germany; bjoern.schumacher@uni-koeln.de

**Keywords:** p63, p73, p53 family, CEP-1, tetramerization, transcriptional activity, oocyte death, development, quality control

## Abstract

The production of haploid gametes through meiosis is central to the principle of sexual reproduction. The genetic diversity is further enhanced by exchange of genetic material between homologous chromosomes by the crossover mechanism. This mechanism not only requires correct pairing of homologous chromosomes but also efficient repair of the induced DNA double-strand breaks. Oocytes have evolved a unique quality control system that eliminates cells if chromosomes do not correctly align or if DNA repair is not possible. Central to this monitoring system that is conserved from nematodes and fruit fly to humans is the p53 protein family, and in vertebrates in particular p63. In mammals, oocytes are stored for a long time in the prophase of meiosis I which, in humans, can last more than 50 years. During the entire time of this arrest phase, the DNA damage checkpoint remains active. The treatment of female cancer patients with DNA damaging irradiation or chemotherapeutics activates this checkpoint and results in elimination of the oocyte pool causing premature menopause and infertility. Here, we review the molecular mechanisms of this quality control system and discuss potential therapeutic intervention for the preservation of the oocyte pool during chemotherapy.

## 1. Introduction

Quality control processes are essential for every aspect of cellular function. Of particular importance is the surveillance of the genetic information stored in the DNA, as mutations can have severe consequences ranging from dysfunctional cells to the development of cancer. This genetic information is under constant threat from errors in the replication process, from the action of transposons as well as from chemical modifications or irradiation-induced damage. Over millions of years, cells have evolved elaborate mechanisms that proofread newly synthesized DNA and repair chemical alterations to the DNA′s backbone and bases. However, if DNA repair is unsuccessful, cells can induce apoptosis to prevent the development of cancerous cells. The central integration hub determining the fate of damaged somatic cells is the transcription factor p53 which orchestrates both DNA repair pathways as well as apoptosis, cell cycle arrest and metabolic reprogramming, and a large number of studies have investigated the interplay between these pathways [1,2,3,4,5,6].

The effect of mutations is even more severe when they arise in a stem cell, as these mutations are inherited by all progeny cells. The most dramatic effects occur in germ cells, with all cells of the developing body and all subsequent offspring being affected. Because of this importance, germ cells have developed specialized quality control systems that consist of specific checkpoints that monitor the integrity of the DNA as well as the correct alignment of homologous chromosomes during meiosis. In oocytes, the details of these quality control processes have recently been elucidated. At least three different checkpoints have been identified that eliminate oocytes, that fail to fulfil the quality control criteria. The first one, named for meiotic silencing of unsynapsed chromatin (MSUC), is triggered when one or two chromosomes are asynapsed and involves extensive heterochromatinization and transcriptional downregulation [7,8]. Oocyte death occurs as a consequence of critical gene silencing for meiotic progression. The second checkpoint also controls correct synapse of homologous chromosomes, however, gets activated when the degree of asynaptic chromosomes is much higher [7,9]. The third checkpoint initiates apoptosis when DNA damage, in particular DNA double-strand breaks (DSBs), is detected. While the first two mechanisms eliminate oocytes with defects in the meiotic process, the third one eliminates oocytes with DNA damage either due to meiotic recombination or caused by other endogenous or exogenous factors. Investigation of this third checkpoint has revealed the mechanism by which female cancer patients treated with chemotherapy become infertile [10,11,12,13,14,15] due to its activation triggered by DNA damage inflicted by γ-irradiation or DNA damaging chemicals (e.g., chemotherapeutic drugs). Here, we review the molecular mechanisms, with a focus on the DNA damage checkpoint, show its evolutionary conservation and discuss the potential for fertility-saving therapies.

## 2. Double-Strand Break Checkpoint

Despite the high demand for the structural integrity of the DNA, the development of fertilization-competent oocytes starts with a massive insult to the cell’s DNA. Before entering meiotic division and formation of gametes, oocytes align their homologous chromosomes in zygotene stage of prophase I. Once aligned, the type II topoisomerase-like DNA transesterase Spo11 promotes hundreds of DSBs per cell, which induces the exchange of genetic material between paired chromosomes by the crossover mechanism [16,17]. This massive induction of DNA DSBs, of course, requires an efficient repair mechanism. Following the exchange of genetic material between homologous chromosomes, lesions are repaired by the homologous recombination (HR) pathway that is dependent on DMC1 and RAD51 [18]. Approximately 10% of the lesions are thus repaired by a crossover and 90% by a non-crossover mechanism. In the following diplotene phase, chromosomes start to separate but remain attached by chiasmata. Around the time of birth (embryonic day 18.5 (E18.5) to five days after birth (P5) in mice), oocytes enter dictyate arrest and remain in this phase until rising levels of luteinizing hormone initiate their reentry into the cell cycle and recruit oocytes for ovulation [19].

The quality control system of the oocyte contains, in addition to the repair system, a second layer: all oocytes that are unable to repair the DNA DSBs following the process of homologous recombination are eliminated by apoptosis. The removal of damaged oocytes is tightly linked to the expression of TAp63α [20,21]. The protein p63 is a member of the family of the tumor suppressor protein p53 and is expressed as multiple isoforms in different tissues [22]. These isoforms are created by the use of two different N-terminal promotors that create either proteins containing the full-length transactivation domain (TA-isoforms) or with a truncated transactivation domain (ΔN-isoforms). The different N-termini are combined with several different C-termini created by splicing events [22,23,24]. The expression of TAp63α, the longest and only expressed isoform in oocytes, increases around the time of birth, with approximately 20% of oocytes showing expression at E18.5 [25]. At P5, virtually all oocytes show strong TAp63α expression [20]. The expression of TAp63α coincides with the time when oocytes have finished the process of homologous recombination and have repaired the Spo11 induced DSBs. All oocytes that have failed to repair their chromosomes get eliminated via the induction of apoptosis. Of the approximately seven million oocytes that are created during embryonic development in humans, only two million survive this process and are present at the time of birth [14,26,27]. In addition to the meiosis-related DSBs, DNA damage is also caused by the action of long interspersed element 1 (LINE-1) retrotransposons, which become activated during the epigenetic reprogramming of embryonic germ cells [28]. During the entire dictyate arrest phase, the expression level of TAp63α remains high; growing oocytes, however, lose TAp63α expression [20]. Experiments with mice have demonstrated a direct connection between the expression of TAp63α and the induction of apoptosis following DSBs: γ-irradiation with 0.45 Gy resulted in the elimination of virtually all primary oocytes, while secondary and antral oocytes, which do not express p63, survive [20].

The mechanism of induction of apoptosis by TAp63α has been described in several studies. The two BH3-only proteins, PUMA and NOXA, are direct transcriptional targets of p63 [29]. Their combined effect of inhibiting the pro-survival family member Bcl-2 [30,31] and activation of pro-apoptotic family member Bax results in oocyte death. The involvement of the entire pathway has been demonstrated with selective knock-out mouse models [29,32]. Inactivating TAp63α in mice renders them resistant to γ-irradiation-induced oocyte death [20]. Likewise, oocytes of the PUMA^−/−^ mouse, and in particular of the PUMA^−/−^ NOXA^−/−^ mouse, are not affected by γ-irradiation [29]. Finally, inactivation of the pro-apoptotic Bcl2 family member Bax also rescues primary oocytes despite γ-irradiation-induced or persistent meiotic DNA DSBs [33] (for more details on these mouse studies, see below).

## 3. Evolutionary Conservation of Genetic Quality Control in Oocytes

**Figure 1:**
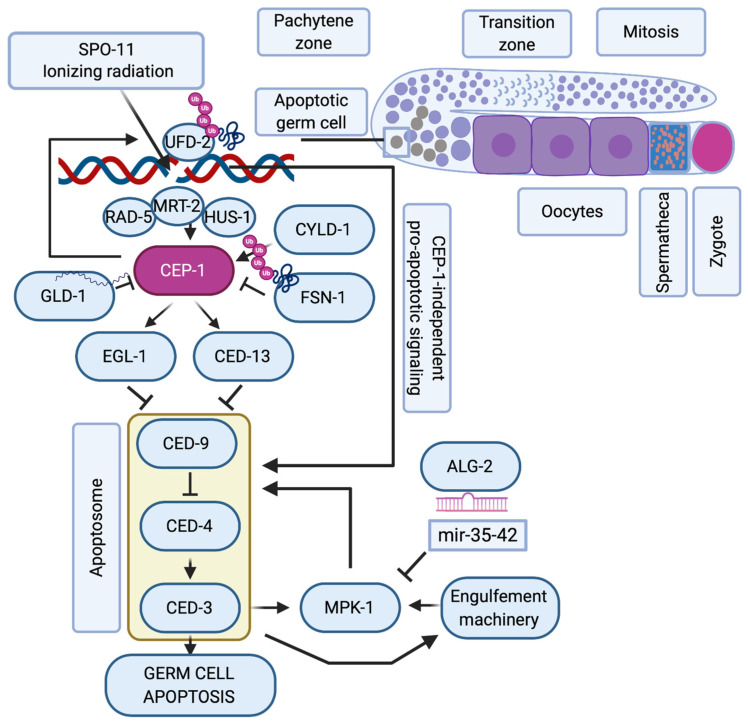
*C. elegans* p53-like, CEP-1, regulates the apoptotic response to DNA damage during oogenesis. Schematic representation of one arm of the nematode’s germline where mitotic germ cells transit into meiosis. When SPO-11 or ionizing radiation (IR)-induced double-strand breaks persist into late pachytene, the DNA damage checkpoint proteins RAD-5, MRT-2 and HUS-1 induce the activation of CEP-1, that is available only at the late pachytene stage of meiosis when its mRNA translation is no longer repressed by GLD-1. CYLD-1 and FSN-1 regulate CEP-1 stability. CEP-1 transcriptionally induces EGL-1 and CED-13 that alleviate CED-9 sequestration of CED-4, which then ignites the caspase CED-3. The regulation of germ cell apoptosis is further fine-tuned by CEP-1-dependent formation of UFD-2 regulated ubiquitin hubs that promote apoptosis only after resolving the repair machinery amid unrepairable DSBs. When the apoptosome and the engulfement machinery are initiated, they trigger the activation of the ERK-MAPK MPK-1 axis, whose activity is controlled via the ALG-2 regulated microRNAs mir-35-42. Only when MPK-1 is activated, is the cell fully committed to undergoing apoptosis, while other pachytene cells continue oogenesis until they are fertilized in the hermaphrodite’s spermatheca to form a zygote.

While there has been considerable evolution of the p53 structure and divergence into p53, p63, and p73 [34], the role of the p53 family in regulating the DNA damage response during oogenesis has evolutionary remained highly conserved and is probably the most ancestral function of this family [35]. Many molecular details of this pathway could thus be elucidated in model organisms such as *Caenorhabditis elegans* and *Drosophila melanogaster.* In *C. elegans,* the p53-like protein CEP-1 regulates the cell cycle arrest of primordial germ cells upon DNA damage [36], and DSBs in meiotic pachytene cells lead to CEP-1 mediated programmed cell death during oogenesis [37,38] (Figure 1). In this organism, germ cells are protected from cell death during the phase of SPO-11 induced DSBs through repression of *cep-1* mRNA expression by the Quaking-like protein GLD-1 [39]. Only when meiotic recombination intermediates or exogenously induced DSBs persist into late meiotic pachytene, CEP-1 protein becomes available and induces the expression of the BH3-only domain proteins EGL-1 and CED-13, that then trigger apoptosis by inhibiting the Bcl2-like CED-9 [40,41]. The recombination repair process itself impinges on the cell death regulation and was proposed to hold off apoptosis until UFD-2-mediated ubiquitin hubs turn over the DSB repair complex at persistent damage sites [42]. The stability of CEP-1 in pachytene cells is also directly regulated by ubiquitin-dependent protein degradation, namely the SCF FSN-1 ubiquitin ligase [43] and the deubiquitinase CYLD-1 [44]. The apoptotic response to meiotic DSBs during oogenesis requires additional regulation by the ERK1/2 mitogen-activated protein kinase MPK-1 [45,46] that regulates the cell death decision upon initial triggering of the apoptotic machinery [47]. The CEP-1-mediated apoptotic demise of genomically compromised pachytene cells is thus a highly regulated and fine-tuned mechanism to ensure the inheritance of stable genomes.

While the domain composition of CEP-1 that contains a Sterile Alpha Motif domain (SAM, a domain type implicated in protein-protein interactions) shows that it is evolutionarily more closely related to p63 than to p53, the p53-like protein from *Drosophila*, Dmp53 [48,49,50], lacks this SAM domain [34] but is expressed in several isoforms with differing potential to induce apoptosis [51]. Dmp53 is expressed in several organs of the fly [52,53] including the primordial germ cells [54,55,56], where it gets activated by genotoxic stress. Cell death following IR-induced DNA damage is initiated by the phosphorylation of Dmp53 by MNK, the *Drosophila* CHK2 homolog [57,58] and Dmp53-dependent expression of the proapoptotic target genes *HID*, *REAPER* and *SICKLE,* of which *HID* seems to play a central role [57].

## 4. Molecular Mechanism of Activation of TAp63α

In contrast to the situation in invertebrates, the very long arrest phase of oocytes in mammals (more than 50 years in humans), in combination with the high expression level of the pro-apoptotic transcription factor TAp63α, requires very tight control of its transcriptional activity. In uncompromised oocytes, TAp63α is inactive by adopting a closed and compact conformation [59]. In principle, all members of the p53 protein family are tetramers due to the presence of a specialized oligomerization domain [60,61] (the only currently known exception is the *C. elegans* protein CEP-1, which forms only dimers [34]). This domain facilitates the formation of tetramers by two different interfaces. The first interface that is used to form dimers mainly consists of one β-strand from each monomer that interact with each other in an anti-parallel manner. An additional helix per monomer stabilizes this arrangement. The interaction of two dimers forms tetramers via these helices. In p63 and p73, the tetramer is further stabilized by a second helix per monomer, which reaches across the tetramer interface [62,63]. In contrast to all other members of the p53 protein family, TAp63α is kept in its inactive conformation in only a dimeric state [59]. Inhibition is achieved by the formation of a six-stranded antiparallel β-sheet [64] consisting of the transcriptional inhibitory domain (TID) [65] of each monomer and two β-strands formed from sequences near the N-terminal transactivation domain. This β-sheet most likely blocks the tetramerization interface of the oligomerization domain, thus enforcing only a dimeric state [64]. Interestingly, one of the β-strands of the inhibitory β-sheet provided by the N-terminal segment corresponds to the second part of the bipartite transactivation domain of p53 [66] and p73 [67], which are unable to form a closed dimeric state [68] suggesting that, during evolution, an inhibitory element (the β-strand of p63) has been converted into an activation element (an α-helical segment in p53 and p73) [69].

**Figure 2:**
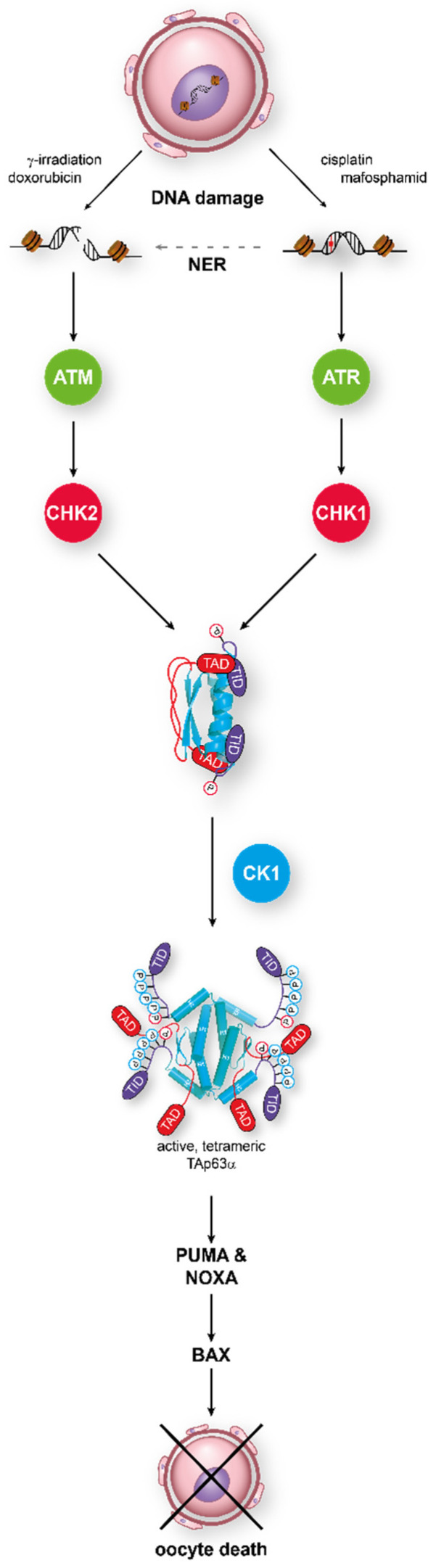
TAp63α acts as the central integrator for DNA damage signaling in oocytes. Detection of either double-strand breaks or single-strand breaks activates the ATM/CHK2 or the ATR/CHK1 pathway, respectively. Phosphorylation of TAp63α on S582 by CHK2 or CHK1 recruits a second kinase, CK1, which adds four more phosphates. This leads to opening of the closed dimeric state. In its active tetrameric conformation, TAp63α acts as a transcriptional activator resulting in the expression of PUMA and NOXA which, in turn, activate Bax.

In this dimeric state, TAp63α is stable until DNA damage—in particular, DNA DSBs—is detected. This triggers the activation of a kinase cascade starting with Ataxia Telangiectasia Mutated kinase (ATM), which phosphorylates histone H2AX to mark the position of the lesion and also activates the Checkpoint kinase 2 (CHK2) [70]. In an elegant study, it was shown that TAp63α is a direct target of activated CHK2, phosphorylating S582, a residue that is situated in a loop between the SAM domain and the TID [71]. This phosphorylation by CHK2, while necessary, is not sufficient for tetramerization and activation of p63 in oocytes [64,72]. Instead, S582-phosphorylated TAp63α becomes a target for another kinase, Casein kinase 1 (CK1). This kinase requires pre-phosphorylated targets with the consensus sequence pS/T-x-x-S/T, where pS/T is either a phosphorylated serine or threonine residue and x any amino acid [73,74]. In TAp63α, a stretch with four serines/threonines showing this pattern follows S582 and is phosphorylated by CK1 (Figure 2). Mutational analysis has revealed that the third phosphorylation event is the most critical one for the activation process [72]. Mutating this third serine (S591) to alanine abrogates activation of TAp63α, while mutating the fourth residue, T594, to alanine only slows down activation. Interestingly, measuring the phosphorylation kinetics has demonstrated that it is this third phosphorylation that is ~20 times slower than the kinetics for the first two modifications [75]. Mechanistically, the slow third step defines the level of DNA damage that is necessary to kill the oocyte by enabling oocytes with low damage to survive by fast degradation of the activated TAp63α.

**Figure 3:**
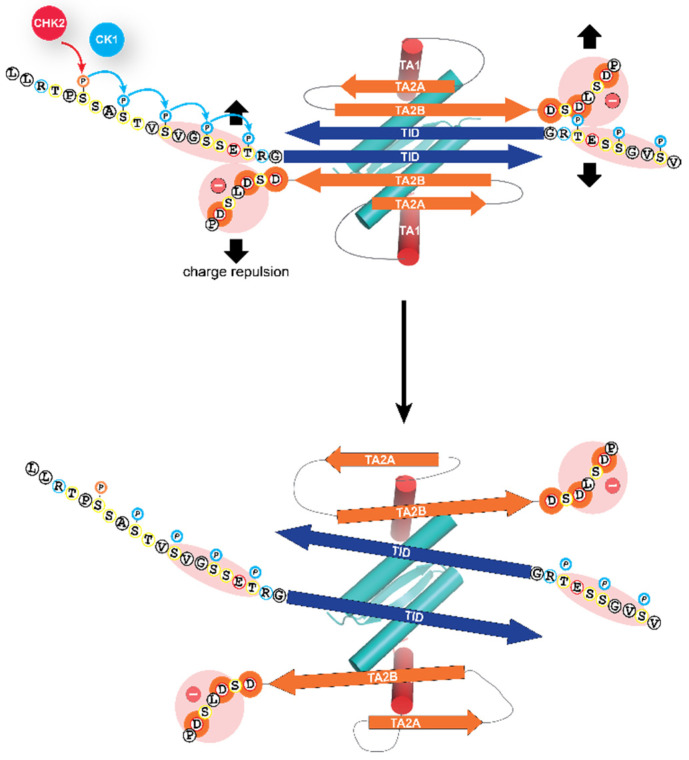
Molecular mechanism of the activation process. The closed dimeric state of TAp63α is stabilized by the formation of a six-stranded antiparallel β-sheet, formed by the C-terminal TI domain and two segments close to the N-terminal transactivation domain, TA2A and TA2B. This β-sheet blocks the tetramerization interface of the oligomerization domain. The addition of the phosphate groups by the kinase CK1 results in electrostatic repulsion with a negatively charged cluster of aspartic acid residues leading to the opening of the closed dimeric state. The segment labeled TA1 forms a helix that in the closed conformation occupies the same site on the oligomerization domain as the second helix of the oligomerization domain in the full tetrameric state [62,64,76]. Only the TA1 peptide sequence acts in the activated state as a transactivation domain, while the two β-strand sequences TA2A and TA2B act as elements of the inhibitory mechanism, thus differing from the bi-partite transactivation domains of p53 and p73 [69].

Phosphorylation of these amino acids directly N-terminal to the TID results in the opening of the closed, dimeric conformation, by electrostatic repulsion with a negatively charged stretch of amino acids located C-terminally to the second β-strand of the inhibitory six-stranded β-sheet (Figure 3). This opening has two effects: it unmasks the tetramerization interface of the oligomerization domain, thus enabling the formation of tetramers for high-affinity DNA binding [64] and it also unmasks the N-terminal transactivation domain making it possible for TAp63α to interact with p300/CBP [69].

The importance of this activation mechanism is also seen in female patients suffering from primary ovarian insufficiency (POI) due to insertions or deletions in the p63 gene. In patients with a heterozygous two nucleotide deletion (delTT1576) in the C-terminus of p63, the entire ovary is absent [77], as is the case with two sisters with a nonsense mutation in the TID of p63 [78]. Two other female patients with another C-terminal deletion (delC1783) suffer from premature menopause around the age of 30 years [79]. Recently, patients with POI were described with nonsense mutations in the C-terminal part of the SAM domain (R555*, W559*) [80,81] and with an intragenic duplication in *TP63* [82]. The common mechanism of all these mutations is that the resulting p63 protein lacks the inhibitory TID, thus creating a constitutively active form of TAp63α which induces oocyte death even in the absence of DNA damage [59]. This mechanism was confirmed in a very recent mouse study in which female mice heterozygous for the alpha-C-terminus were completely infertile, showing a ~40% reduction in primary oocyte number at P1 and a complete absence of oocytes at P10 (Lena et al., NatCom, in press). Several human syndromes are caused by mutations in the p63 gene, which are manifested by craniofacial, limb and ectodermal development disorders [83,84,85,86,87,88]. It is predicted that female patients with p63 mutations will suffer from infertility if the mutations create constitutively active p63 forms (for example, by frame shift or nonsense mutations in the C-terminus which delete the TID) that effectively kill all oocytes (Lena et al., NatCom, in press) [77,79,80,81]. In contrast, mutations in the DBD (causing the Ectrodactyly-Ectodermal dysplasia-Clefting (EEC) syndrome [83,84,89]) prevent the binding to DNA, which compromises the p63-based quality control function. Mutations in the SAM and TID domains, that cause the Ankyloblepharon-Ectodermal dysplasia-Cleft lip/palate (AEC) syndrome [88], can also lead to the formation of open TAp63α forms that, in principle, kill affected oocytes. However, AEC-related mutations result in the exposure of aggregation prone peptide sequences followed by aggregation of p63 [90], which compromise the quality control system in oocytes (Lena et al., NatCom, in press). As a result of these two opposing effects (formation of open and tetrameric forms, inactivation through aggregation), premature ovarian insufficiency during early adulthood has been diagnosed in some affected patients [80,81].

While the general role of the p53 protein family in monitoring the genetic quality in germ cells is conserved from *C. elegans* to humans, the exact activation mechanism described above seems based on sequence comparisons only conserved in vertebrate species [72,75]. CEP-1 is a constitutive dimer while the Drosophila protein Dmp53 is a constitutive tetramer [34]. Nevertheless, both get activated by phosphorylation as described above, however, without changing their oligomerization status.

## 5. Additional Activation Mechanisms

The DSBs induced activation constitutes the most efficient way to activate TAp63α. However, it was also shown that activation follows not only the ATM-CHK2 pathway but also the Ataxia Telangiectasia and Rad3-related kinase (ATR)-Checkpoint kinase 1 (CHK1) route that is typically activated by single-strand DNA breaks [91]. The same result was obtained in studies of mouse oocytes defective in either meiotic DSB repair (Trip13^Gt/Gt^) or in Spo11^−/−^ oocytes, which are unable to align chromosomes properly and suffer from DNA damage (see below). Inactivation of CHK2 did not rescue the entire oocyte pool, but only ~33–25% compared to the wild type level. This suggested that an additional pathway for the activation of TAp63α might exist and further experiments confirmed that, in the absence of CHK2, CHK1 gets activated [92]. Activation of CHK1 was also seen in the treatment of mice with cyclophosphamide [93]. At 24 h after treatment of mouse ovaries with 4-hydroxyperoxycyclophophamide (4-HC, an active metabolite of cyclophosphamide), phosphorylated ATR, CHK1 and CHK2, but no activated ATM, was detected. This is consistent with the molecular mechanism of 4-HC that induces DNA crosslinks rather than DNA DSBs, which results in the activation of ATR rather than ATM. The use of ATR or CHK2 inhibitors protected primary oocyte from 4-HC-induced loss, suggesting that this chemotherapeutic agent induces the ATR/CHK2/TAp63α pathway. In general, activation of CHK2 by ATR following ionizing radiation has been documented in cell lines, and it was demonstrated that this cross-talk is enhanced if ATM is inhibited [94].

Earlier studies had also suggested that the kinase c-Abl is responsible for the activation of TAp63α and inducing oocyte death [95]. However, inhibitor studies [72], as well as genetic inactivation of both c-Abl1 and c-Abl2 in mice [91], demonstrated c-Abl is not involved in the direct activation mechanism. These results were also confirmed in human ovarian xenograft studies in nude mice [96].

## 6. Other Family Members Involved in Oocyte Death

Mouse studies have demonstrated that the main member of the p53 protein family responsible for the surveillance of the genetic integrity in oocytes is p63 [20]. The first full p63 knock-out mouse did not reveal the involvement of p63 in oocyte quality control, as p63 is also highly expressed in the basal layer of stratified epithelial tissues [97]. Abrogation of the expression of the p63 isoform in these tissues resulted in mice showing severe developmental defects, including limb truncations and lack of a multi-layered skin and other epithelial structures [98,99]. In contrast to the complete p63 knock-out mouse, the TAp63 isoform-specific knock-out mouse develops normally, however, it lacks the quality control in oocytes. Inflicting DSBs with γ-irradiation eliminates, in wild type mice, all primary oocytes within two days, while they survive in TAp63^−/−^ mice. The same treatment of p53^−/−^ mice results in the elimination of all primary oocytes [20], showing that TAp63α is the central factor inducing apoptosis in response to DSBs. However, when DNA repair is compromised, and DSBs persist, p53 seems to become activated and induce apoptosis. This mechanism was shown in mice in which inactivation of TAp63α initially rescued primary oocytes following γ-irradiation with 0.45 Gy but resulted in the complete elimination of the entire pool at seven days post-treatment [71]. The double knock-out p53^−/−^TAp63^−/−^ preserved the primary oocyte pool to a similar extent as seen in CHK2^−/−^ mice. Interestingly, CHK2 is required for both p53 and TAp63α activation. These results are consistent with a recent study with TAp63^−/−^ mice. Using inhibitors of RAD51 prevented the HR-based repair of DSBs following γ-irradiation with 0.45Gy. This treatment resulted in depletion of primary oocyte numbers and increased apoptosis of oocytes despite the lack of TAp63α [100]. The same study reported that large numbers of mature oocytes could be ovulated in TAp63^−/−^ mice exposed to 0.1 Gy, however, only low numbers could be ovulated when these mice were exposed to 0.45 Gy. These mice were irradiated at P10 but allowed to mature to adulthood before hormonal treatment to obtain oocytes. This unexpectedly low number might be due to p53-induced oocyte apoptosis, which would also be consistent with the different activation time intervals of TAp63α and p53 with TAp63α being activated quickly and p53 when DSBs persist over more extended periods. Such a model could also explain studies showing that p53 is not necessary for oocyte death induced by doxorubicin or γ-irradiation [20,29,101] because of the different time scales used in these experiments.

The third family member, p73, does not seem to play a major role in this first checkpoint of oocyte quality control. p73 is more similar in its function to p53, acting as a tumor suppressor, but has additional developmental roles in neuronal development and in maintaining transcriptional programs in ciliated epithelial tissues [102,103,104]. However, both sexes of mice in which the TAp73 isoform has been inactivated are infertile despite their normal mating behavior, i.e., they have no defects in pheromonal pathways as described for the p73^−/−^ mouse [102], and female mice show normal cyclicity [105,106]. In females, this infertility was shown to be caused by ovulated oocytes being trapped under the bursa and not being able to migrate towards the fallopian tube [105]. Investigation of in vitro matured germinal vesicles and ovulated oocytes revealed a high level of spindle abnormalities, such as multipolar spindles, spindle relaxation and scattering. Furthermore, in vitro fertilization of TAp73^−/−^ oocytes resulted in high numbers of embryos with multinucleated blastomeres and in blastocysts with an abnormal cell number. These results suggested a link between TAp73 and the spindle assembly checkpoint, which regulates the correct attachment of sister chromatids both to the mitotic and meiotic spindle [106]. Indeed, in oocytes from TAp73^−/−^ mice, several components of the spindle assembly checkpoint were found to be mislocalized, including the mitotic kinases Bub1 and BubR1 [106]. The same study also demonstrated a direct physical interaction between TAp73α and Bub1, Bub3, as well as BubR1. The exact molecular interactions between TAp73α and components of the spindle assembly complex and its potential contribution to quality control mechanisms, however, have yet to be studied in detail.

The direct involvement of all three family members was proposed in a different study [107]. These investigations also showed that TAp63α is the master regulator of cisplatin-induced oocyte cell death. However, according to that model TAp63α controls the expression of the kinase c-Abl, of TAp73 and potentially of p53. c-Abl phosphorylation of TAp73 leads to the expression of Bax, initiating apoptosis. However, in this study, several results, including the apparent toxicity of cisplatin not only to primary oocytes but also to somatic ovarian cells, could not be explained [107,108].

## 7. Chromosomal Synapsis Checkpoint

**Figure 4:**
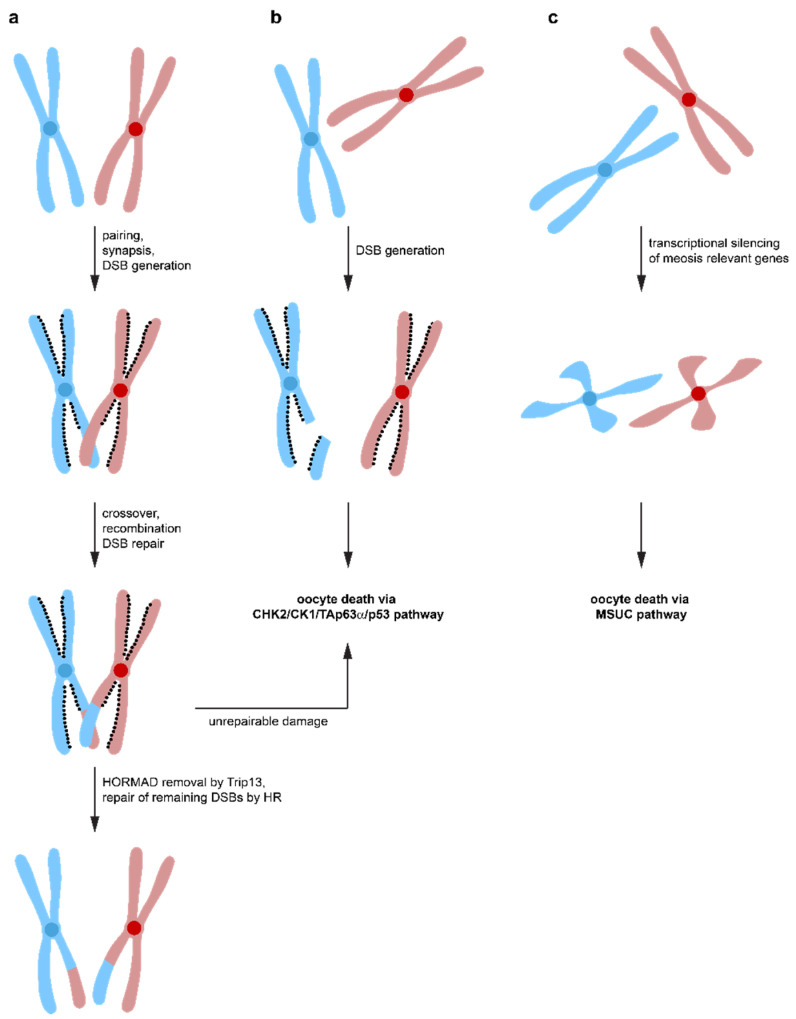
Different pathways lead to oocyte death. (**a**) During the zygotene stage of meiosis, homologous chromosomes synapse to form aligned pairs. Spo11 induces DNA DSBs that lead to crossovers between homologous chromosomes, which is required for stable synapsis. Repair by homologous recombination results in exchange of genetic material. DNA repair via inter sister-chromatid homologous recombination is suppressed by the HOMRAD1 proteins lining the chromosome axes. After successful DNA repair, Trip13 removes HOMRAD1 proteins, enabling DNA repair to occur via the inter-sister chromatid pathway. If DNA damage is detected that cannot be effectively repaired, oocyte death is initiated via the CHK2/CHK1/TAp63α pathway. (**b**) failure to align homologous chromosomes correctly, e.g., in Spo11^−/−^ oocytes, inhibits DNA repair by homologous recombination using the homologous chromosome as a template. At the same time, inter-sister chromatid repair is suppressed by the HOMRAD1 proteins. The persistent DNA DSBs lead to activation of the CHK2/CHK1/TAp63α pathway and oocyte death. (**c**) if a small number of chromosomes is not correctly aligned, heterochromatinization leads to suppression of transcription and oocyte death via the MSUC pathway.

Spo11-induced DSBs not only constitutes a major challenge for the oocyte′s DNA repair system, but surprisingly, it is also required for correct oocyte progression through meiosis. Spo11^−/−^ female mice are infertile due to defects in chromosome synapsis. DSBs are a prerequisite for the formation of crossovers between homologous chromosomes, which enables the spindle apparatus to recognize these chromosome pairs as a single entity [109,110,111]. Failure to align chromosomes correctly results in aneuploid germ cells causing either congenital disorders or pregnancy loss when they remain undetected and a reduced amount of crossover between chromosomes correlates with increased aneuploidy ranging from yeast to humans [112,113,114,115]. In Spo11-deficient female mice, asynaptic chromosomes suffer from a high level of spontaneous DSBs, probably from LINE-1 retrotransposon activation during early pachynema [28,116]. These spontaneous DNA lesions would usually get repaired via the HR pathway using the homologous chromosome with which the damaged chromosome is aligned. Repair via the sister chromatid is suppressed by the presence of HORMAD1/2 (Hop1, Rev7, and Mad2 domain-containing proteins) that bind to the axes of meiotic chromosomes in early prophase I [117]. Inactivation of HORMAD1 results in infertility due to failure in pairing and synapsis of chromosomes [118], however, the number of oocytes was not affected [119] suggesting that HORMAD1 is part of a synapsis checkpoint. Likewise, the inactivation of HORMAD1 rescued the massive loss of oocytes in Spo11^−/−^ mice. Inactivation of HORMAD2 in oocytes with a Trip13 mutational background even rescued the fertility [120]. Interestingly, the elimination of oocytes with a high number of asynapsed chromosomes depends on CHK2, and inactivation of CHK2 in Spo11^−/−^ mice rescues a large number of oocytes (but not all, probably due to activation of the ATR/CHK1 pathway, see above) [120]. Overall, these data suggest a model in which Spo11 induced DSBs are necessary to form crossovers between homologous chromosomes to ensure accurate chromosome pairing. These DSBs get repaired by the HR pathway using the homologous chromosomes as templates (Figure 4). Repair via the inter sister chromatid is initially suppressed by HORMAD1/2 [121,122,123,124,125]. After correct synapsis, HORMAD1/2 are removed from the chromosomes (in a Trip13-dependent manner) and further repair of DSBs by the inter sister chromatid pathway is possible, probably contributing a significant amount of the total DNA repair [120]. In case a small number of asynapsed chromosomes exist, the transcriptional silencing of these chromosomes triggers oocyte death by silencing genes essential for meiotic progression (MSUC mechanism). If the number of asynapsed chromosomes is larger (more than 1–3 chromosomes), the accumulation of DSBs on these chromosomes triggers the CHK2/CK1/TAp63α/p53 pathway for the elimination of these oocytes. DNA repair on these asynapsed chromosomes is not possible due to the absence of the homologous chromosome and the inhibition of the inter sister chromatid repair pathway by the presence of HORMAD1/2. As a result, only one checkpoint, the DNA damage checkpoint, seems to exist, triggered both by non-repaired DSBs originally induced by Spo11 and by the accumulation of DNA damage on asynapsed chromosomes. While the first mechanism is triggered before or at the beginning of the dictyate arrest phase, the second mechanism can be triggered at any time, dependent on when the accumulated DNA damage reaches the critical threshold.

## 8. Chemotherapy-Induced Infertility in Female Cancer Patients

The knock-out mice studies, cell culture experiments and biochemical investigations summarized above have clearly demonstrated the role of TAp63α as the master regulator of a genetic quality control program in primary oocytes and the mechanism of its regulation via its oligomeric state. These results have also provided the mechanism that causes infertility in human female cancer patients and patients treated for sickle cell anemia or certain autoimmune diseases with chemotherapy and/or γ-irradiation. Drugs such as cisplatin, doxorubicin or cyclophosphamide damage the DNA, trying to induce apoptosis in fast-dividing cells with the intention of eliminating tumour cells. Due to the non-cell-type selectivity of these chemotherapeutic drugs, they also damage DNA in oocytes, resulting in the activation of TAp63α. This unique TAp63α-based quality control system makes the elimination of damaged oocytes very efficient. In mice, a tight dose–response curve has indeed been observed in irradiation experiments. While most oocytes in P4 mice survived irradiation with 0.1 Gy (~three DSBs per cell), virtually all primary oocytes were eliminated by 0.45 Gy (~ten DSBs per cell) [20]. In humans, the LD_50_ total body irradiation dosage for the loss of the primary oocyte reserve was extrapolated to be less than 2 Gy, while the typical total body irradiation dose for acute leukemia patients is 12 Gy [126,127]. This LD_50_ dose, however, depends also on the age of the patient. Significantly higher irradiation levels are tolerated at young age while older patients are at higher risk of POI, probably mainly due to the lower number of primary oocytes still present [128]. With the increasing survival rate of cancer patients, the quality of life after therapy becomes an important aspect. As the number of oocytes is fixed and new oocytes are not produced after birth, the elimination of all primary oocytes through chemotherapy-induced activation of TAp63α results in premature ovarian insufficiency. In addition to infertility, the loss of the oocyte pool causes a breakdown of the endocrine function of the ovary, which can lead to further health impairments such as osteoporosis, higher risk for certain cancer types as well as heart and psychological problems [13,14,129]. This problem is particularly severe for the treatment of prepubertal girls for childhood cancers. The prognosis for many of these childhood cancers is good, with high survival rates [130], but the consequences can be very severe [131,132], and currently, the only option is the surgical removal of (parts of) the ovaries, cryostorage and re-implantation after the end of the therapy [133,134]. However, this procedure bears the risk of also re-introducing tumor cells and surgery might not be possible before the start of chemotherapy, necessitating the search for alternatives to preserve the primary oocyte pool. With the elucidation of the mechanism of inhibition and the activation pathway of TAp63α, pharmacological approaches to inhibit the induction of oocyte death have become possible. At the center of such approaches are the three kinases, ATM, CHK1/2 and CK1, that play essential roles in this activation process. Inhibitor studies in mouse ovaries have indeed shown that using inhibitors for one of these kinases suppresses activation of TAp63α [64,135], allowing oocytes to survive [72,136].

## 9. Fertility Mouse Experiments

Enabling oocyte survival would clearly preserve fertility and the endocrine function of the ovaries and thus enhance the quality of life of female cancer patients post-therapy. However, a central question that has to be answered before the development of an oocyte survival therapy is how efficiently DNA gets repaired following chemotherapy when oocyte death is suppressed. Several studies have started to shed light onto this important question by investigating the health of the offspring of mice with suppressed oocyte apoptosis. In the first study, Kerr et al. have shown that inactivation of PUMA and, even more effectively, of both PUMA and NOXA preserves fertility despite high levels of irradiation (up to 4.5 Gy in that study) [29]. Control groups of wild type mice, NOXA^−/−^ or even mice with a complete NOXA knock out, and only one functional allele of PUMA (NOXA^−/−^ PUMA^+/−^) lost all primary oocytes. Similarly, mice lacking two other BH3-only pro-apoptotic Bcl-2 family members, Bim or Bmf, were not protected. After waiting for seven weeks following γ-irradiation with 0.45 Gy to ensure that all non-primary oocytes had been removed from the ovaries either by atresia or ovulation and that fertilized oocytes must, therefore, be produced from the pool of rescued primary oocytes, female mice were mated with non-irradiated wild type or PUMA^−/−^ males. Out of 16 PUMA^−/−^ females, 13, and out of 12 NOXA^−/−^ PUMA^−/−^ females, nine were fertile, with most of them also producing up to four litters. Investigating the health status of mice born in the first or second litter did not show any differences to mice born to either non-irradiated PUMA^−/−^ or wild type female mice.

To take these investigations further, 14 female mice from the F1 generation were mated with 13 becoming pregnant. A total of 241 offspring of the F1 and F2 generation were studied beyond weaning, and no increase in developmental defects relative to wild type mice of the same strain was noted. Several of the offspring were kept for more than 400 days, but none of these animals developed cancer or showed other abnormalities [29].

Mouse fertility investigations were also performed with chemotherapeutic reagents and showed a slightly different picture [32]. Treating adult wild type mice with cyclophosphamide or cisplatin resulted in almost complete elimination of or a significant reduction in the number of primary oocyte, respectively. Virtually complete rescue from cell death was observed in the PUMA^−/−^ mouse for both chemotherapeutics. When comparing the age of the last pregnancy and the total number of pups born to female mice treated with either a saline solution, cyclophosphamide or cisplatin, a reduction in the total fertile reproductive time of cyclophosphamide-treated mice relative to saline-treated mice was observed (191.6 ± 6.1 vs. 365.2 ± 16.6), with an almost equal number of pups per litter. Remarkably, for the cisplatin-treated female mice, no differences relative to the saline-treated cohort were measured despite a 73% reduction in the number of primary oocytes. This group continued to study the health of pups born to PUMA^−/−^ and TAp63^−/−^ mice treated with the two chemotherapeutics or saline. The only difference between the groups of pups noticed was a reduction in the weight of the pups from cyclophosphamide-treated mice at weaning, which, however, disappeared at day 33.

Another study investigated the involvement of Bax in the elimination of oocytes with irradiation-induced DSBs [33]. They showed that the number of primary oocyte in Bax^−/−^ females following irradiation was equivalent to the number in non-irradiated females and slightly higher than in wild type animals. The litter size of Bax^−/−^ females was slightly decreased compared to non-irradiated Bax^−/−^ and Bax^+/−^ mice. The authors of the same study also investigated the effect of persistent DSBs due to deletions in components of the DNA repair machinery. The meiosis-specific recombinase DMC1 is important in early recombination, by coating resected DSBs to facilitate single-strand invasion and recombination repair [137,138], while MSH5 acts later to stabilize Holliday junctions prior to these late recombination intermediates getting resolved [139,140]. The persistence of DSBs in Dmc1^−/−^ and in MSH5^−/−^ mice leads to the almost complete elimination of all types of oocytes by P21 [33]. Removing PUMA (Dmc1^−/−^PUMA^−/−^ and MSH5^−/−^PUMA^−/−^) or NOXA (Dmc1^−/−^NOXA^−/−^ and Msh5^−/−^NOXA^−/−^) as well did not protect the oocytes, but the double knock-out (Dmc1^−/−^PUMA^−/−^NOXA^−/−^ and Msh5^−/−^PUMA^−/−^NOXA^−/−^) preserved between 30% and 40% of oocytes relative to the PUMA^−/−^NOXA^−/−^ mouse with similar effects seen for the Dmc1^−/−^Bax^−/−^ and the Msh5^−/−^Bax^−/−^ mice. Interestingly, investigating the number of DSBs in surviving oocytes in Dmc1^−/−^PUMA^−/−^NOXA^−/−^ mice showed that RPA2 and RAD51 foci decrease tenfold between P0 and P7, which was interpreted as a sign of DNA repair [33].

Similar results were obtained with CHK2^−/−^ Trip^13Gt/Gt^ female mice, in which inactivation of CHK2 rescues primary oocytes from persistent DSBs [71]. Despite this defect, females became pregnant between four and seven times, albeit with smaller litter sizes. Pups did not show any developmental defects and were monitored for up to 1 year. These results also demonstrated that oocytes have a remarkable capability to repair persistent DNA damage at later meiotic stages if the first DNA damage checkpoint is inactivated.

Finally, Stringer et al. investigated the effect of DNA repair mechanisms in damaged oocytes directly [100]. They observed a dose-dependent increase in nuclear phosphorylated ATM kinase, the occurrence of γH2AX and recruitment of RAD51 to the location of DNA damage. Only 10% of oocytes showed recruitment of DNA-dependent protein kinase catalytic subunit (DNA-PKcs) which suggests that DNA repair in oocytes arrested in prophase of meiosis I occurs predominantly via the homologous recombination (HR) and only a small percentage via the nonhomologous end-joining (NHEJ) pathways. Interestingly, more γH2AX foci were detected in oocytes than in the surrounding somatic cells, which suggests that oocytes have a more sensitive DNA damage response than somatic cells. In addition, DNA repair in somatic cells seems to be predominate via the NHEJ pathway, as seen from the observation of DNA-PKcs recruitment to the location of DNA damage. This result is also consistent with an earlier study that showed that treatment with cyclophosphamide or cisplatin causes DNA damage in primary oocytes and virtually no damage in the surrounding granulosa cells. In contrast, in growing follicles, the somatic cells suffered more DNA damage, while the oocyte showed higher resistance [93,141]. This result can be explained with the loss of TAp63α expression in oocytes in growing follicles, making them more resistant to DNA-damage-induced apoptosis and the fact that rapidly proliferating, mitotically active cells (granulosa cells in growing follicle) are, in general, more sensitive for chemotherapeutic agents than resting cells (granulosa cells in primordial follicle).

Stringer et al. further investigated the timeline of DNA repair in the TAp63^−/−^ mouse [100]. Following the number of γH2AX foci, they showed an almost complete loss of these foci five days after irradiation with 0.45 Gy while 3h post-irradiation 97% of oocytes had shown foci, suggesting that a significant amount of DNA repair occurred. This conclusion was confirmed from mating studies showing that irradiated TAp63^−/−^ mice had a similar litter size to the non-irradiated control group. The authors also evaluated the general health and histological analysis of organs of offspring from irradiated TAp63^−/−^ females. No defects were seen; only a slight reduction in body weight at weaning was observed, possibly due to less efficient nurturing from irradiated females. Finally, the authors used whole-genome sequencing to investigate the overall mutational load of offspring from irradiated TAp63^−/−^ mice but could not see an increase relative to offspring from non-irradiated TAp63^−/−^ mice. Such a result is, on one hand, consistent with the error-free DNA repair pathway by homologous recombination and on the other hand, shows that oocytes have a high capacity for DNA repair when the apoptotic pathway is suppressed.

The importance of DNA repair has recently been demonstrated in mouse studies with the poly(ADP-ribose) polymerase (PARP) inhibitor olaparib which is being used to treat breast cancer patients with germline BRCA1- or BRCA2- mutations. This inhibitor prevents the repair of single strand breaks by PARP [142,143]. Treating mice with this inhibitor showed a significant (36%) reduction in the number of primary oocytes relative to a control group [144]. The exact mechanism of how olaparib treatment results in DNA damage induced primary oocyte death remains elusive. In fast-dividing cancer cells, olaparib causes PARP1 trapping on the DNA, which results in DNA breaks during replication. However, prophase I arrested primary oocytes do not replicate. Whether olaparib causes DNA breaks during transcription or in another pathway is not yet clear. In general, oocyte depletion could be triggered either by activation of TAp63α via an ATR-CHK1 pathway or if single-strand breaks further progress into DSBs by the ATM-CHK2 pathway.

Impaired DSB repair has also been proposed as a mechanism for the age-induced wastage of the ovarian reserve in humans and mice [145]. ATM is not only involved in inducing oocyte death via activation of TAp63α or p53 but is also essential to initiate DNA repair. Oocyte-specific knockdown of ATM in mice resulted in an increase in DSBs and reduced the number of oocytes, similar to knockdown of other repair proteins such as BRCA1, MRE11 or Rad51 [146]. Consistently, germline BRCA1 mutations in women cause a decrease in the ovarian reserve due to impaired DSB repair [146,147], which is accelerated (relative to women without BRCA mutations) by treatment with chemotherapy [148].

Most of these studies focused on DNA DSBs that were created by different methods. A mouse study using cyclophosphamide as an alkylating agent produced different results to the DSB-focused investigations [149]. In the cyclophosphamide-based study with prepubertal mice, a significant effect on the number and quality of oocytes was observed, that got more severe the earlier the mice were treated. The younger the mouse at the time of treatment, the fewer the number of oocytes obtained by superovulation at adulthood. Mice treated at day 14 yielded, on average, 8.5 ± 1.4 oocytes, with 45% of the animals producing no oocytes at all compared to 50.2 ± 3.2 oocytes per animal in the non-treated control group. The percentage of mature MII stage oocytes, however, was similar in both groups. In vitro fertilization rates did not differ between treated and control groups, but in an assay measuring the inner mass proliferation of blastocysts in vitro, the cyclophosphamide-treated blastocysts showed a significant delay that, again, was most pronounced for blastocysts obtained from mice treated at the youngest age. While the reduction in the number of oocytes obtained from superovulation can be explained with TAp63α-induced oocyte death, the defects seen in blastocyst development differ from the other mouse studies [29,32,71,100] that show no significant effects on surviving oocytes. The different assays used in these studies could potentially explain the different results. On the other hand, if confirmed, these results could indicate that alkylating agents and DSB producing agents have different long-lasting effects on oocytes. While oocytes are well prepared for DSB repair by homologous recombination due to the presence of a quadruple set of the genetic material, the repair of alkylated DNA could be less efficient, resulting in longer-lasting effects. This would be consistent with observations in some studies that alkylating agents induce the greatest risk of causing POI [150,151].

## 10. Potential for Suppression of Oocyte Death during Chemotherapy

The genetic mouse studies summarized above have suggested that preventing oocyte death from irradiation/chemotherapy or persistent meiotic DSBs does not result in a significant risk of producing a high number of mutations. This opens an avenue towards the development of a pharmacology-based oocyte protective therapy. A large number of studies have already suggested treatment with a wide variety of different molecules to protect oocytes from chemotherapy-induced apoptosis. These studies range from hormonal treatment with gonadotropin-releasing hormone analogues or luteinizing hormone [152] to the use of sphingosine1-phosphate, dexrazoxane, resveratrol or mTORC inhibitors [153]. Some of these treatments have shown protective effects in mouse models while others, also used in human trials, are controversially discussed. An excellent overview of the different drugs and the results of their use is given in reference [10]. To date, however, none of these investigations has been based on a clear molecular mechanism that could explain the proposed protective function of the suggested molecules. In contrast, the combination of mouse studies and biochemical elucidation of the TAp63α activation mechanisms have provided a scientific basis for developing a rationally designed therapy. Studies with CHK2 inhibitors in mouse ovary cultures have shown that inhibition of this kinase prevents the activation of TAp63α to its tetrameric state [64]. Similarly, ATM and CK1 inhibitors were shown to prevent tetramerization and preserve primary oocytes in mouse ovary culture [72]. In another study, CHK2 inhibitors proved effective in rescuing primary oocytes from γ-irradiation in mouse ovary culture [136]. Re-implanting these ovaries into sterilized host females showed that fertility was protected. In case of treatment of mouse ovary culture with 4-HC, both ATR and CHK2 inhibitors showed protective effects on primary oocytes but not on granulosa cells of multilayer secondary follicles [93]. Similar protective results were demonstrated with an ATM inhibitor in neonatal rat ovarian culture [154].

One problem with a pharmacological intervention to protect oocytes is that the ATM/CHK2/CK1/TAp63α axis is the main, but not the only, way to trigger cell death in oocytes. As mentioned above, ATR/CHK1 can substitute for ATM/CHK2 and not only TAp63α, but p53 also gets activated when DSBs are not efficiently repaired. In addition, several isoforms of CK1 exist that, at least partially, can substitute for each other. The most effective way would be to inhibit the induction of apoptosis directly by targeting Bax, as all pathways converge at that point. However, any fertoprotective therapy should, of course, not compromise the chemotherapy, and induction of apoptosis could be problematic in that respect. Nevertheless, several small molecules have already been tested to alleviate the toxic side effects of doxorubicin. In addition to inducing apoptosis in oocytes, doxorubicin shows cardiomyopathy. A small allosteric inhibitor of Bax that inhibits the insertion of the protein into the mitochondrial membrane has recently been shown to suppress apoptotic and necrotic cardiac cell death without compromising the success of doxorubicin treatment in leukemia or breast cancer models in zebrafish and mice [155,156]. For the same reason (cardiomyocyte protection from doxorubicin), dexrazoxane is already used in the treatment of human cancer patients, where it protects heart cells by sequestering iron ions that otherwise would produce together with doxorubicin reactive oxygen species. Using dexrazoxane also, oocytes were protected in vitro and in vivo [157]. These few examples demonstrate that small molecules can be found that have the potential to protect oocytes without compromising the chemotherapy if used within a certain therapeutic time and concentration window. The (additional) use of inhibitors for the relevant kinases that activate TAp63α and p53 could further enhance the therapeutic possibilities. Cancer is a collection of several hundred individual diseases depending on the affected tissue, mutated gene and exact mutation. Most likely, no single fertoprotective therapy will be used on all patients but depending on the type of cancer (for example, p53-positive or -negative), different ones will be utilized. The detailed molecular elucidation of the TAp63α/p53-based oocyte quality control pathway has provided several new targets that will most likely contribute to the development of such a therapy.

## 11. Conclusions

Most studies to date have shown a high degree of DNA repair capabilities of oocytes that predict that fertoprotective therapies should be safe. This prediction is in agreement with studies with human patients who have been exposed to radiation both for medical as well as non-medical reasons [158] or radiation and alkylating agents during therapy [131,159]. These studies have not found a higher risk of genetic diseases in progeny. Still, more investigations will be required to develop a safe therapy that includes protection not only from ionizing radiation but also from chemotherapy. One difficulty is the transfer of results obtained with mouse studies to human patients. One potential intermediate step on the way to a fertoprotective therapy could be human ovarian tissue transplanted on nude mice. Studies with this system have already been performed [160,161].
